# Supplementary material for: Association between prediabetes and depression: A meta-analysis
Source: PLoS One. 2024 Aug 22;19(8):e0307428. doi: 10.1371/journal.pone.0307428 (PMC11340969; doi:10.1371/journal.pone.0307428)
Supplement: Attachment — Submitted filename: PONE-D-24-07500R1 Reply Letter.docx [file pone.0307428.s001.docx]

Dear Dr. Hennis and the reviewers of Plos One,

Thank you very much for your comments on our manuscript entitled “Association between prediabetes and depression: A meta-analysis” (PONE-D-24-07500). These comments are valuable for improving the quality of our work. We have revised the manuscript accordingly, with changes highlighted in red font. A detailed response letter has also been attached for your reference. Your further consideration is highly appreciated.

Look forward to hearing from you at your earliest convenience.

Best regards,

Corresponding author:

Weitao Wan

Department of Psychiatry, Tianyou Hospital Affiliated to Wuhan University of Science and Technology, No. 9, Tujialing, Dingziqiao Road, Wuchang District, Wuhan, Hubei, China. E-mail: wwt686@sohu.com

Reviewer #1:

(1) The bases for conducting this review and meta-analysis are clear and solid. To better understand how previous studies on the relationship between prediabetes and depression are inconsistent, it is suggested to expand on the idea that they are inconsistent. Furthermore, considering that references 18-33 are studies with populations from various places and methodologies, this aspect could be briefly mentioned.

**Author’s reply:** Thank you for your comments. We have briefly expanded the description of the inconsistent results of the previous studies as suggested in the revised Introduction part as “However, the results of previous studies were not consistent [18-33]. Some of them supported that prediabetes was related to depression [24-27, 29, 30], while other studies did not found a statistically significant association [18-23, 28, 31-33]. In addition, these studies are with populations from various places and of different study definitions and methodologies for evaluating prediabetes and depression [18-33]. It remains unknown whether these factors may influence the association between prediabetes and depression”.

(2) The objective of the review and meta-analysis is clear.

**Author’s reply:** Thank you for your comments.

(3) Methodological guidelines for review and meta-analysis are appropriate (PRISMA and Cochrane). It is striking that the manuscript does not detail whether the review protocol was published in Prospero or elsewhere. Presenting the protocol before data extraction promotes transparency of methods and reduces biases, which can be reviewed, and unnecessary duplication of effort between researchers.

**Author’s reply:** Thank you for your comments. The protocol of the manuscript was not registered prospectively. We have clarified this in the revised manuscript as a limitation of the study.

(4) Regarding the search terms, it is suggested to indicate whether they were compared with the MeSH terms.

**Author’s reply:** Thank you for your comments. The search terms were based on key words of rather than MeSH terms to improve the sensitivity of the database search. However, a comparison with MeSH terms was performed before database search to ensure all relevant MeSH terms are included in the search terms. This has been clarified in the revised Methods part.

(5) Recommends that this entire paragraph be part of the “inclusion and exclusion criteria” (not the literature search): “the search was limited to studies in humans. Furthermore, we only considered studies published as full articles in peer-reviewed journals in English. As a complement, references of related original and review articles were manually examined to identify potentially related studies. Publications published from the inception of the databases to December 8, 2023 were examined.”

**Author’s reply:** Thank you for your comments. We have removed this paragraph from “literature search” to “inclusion and exclusion criteria” as requested.

(6) Given the results of the meta-analysis in the conclusions, it is suggested that prediabetes was associated with a slightly higher prevalence of depression.

**Author’s reply:** Thank you for your comments. We have revised the conclusions in the abstract and the manuscript as suggested to emphasize that prediabetes was associated with a slightly higher prevalence of depression.

(7) The final statement of the conclusions is risky in light of these results since it suggests a causal relationship that the study has not determined. With these results, it is not possible to conclude that disorders of glycemic metabolism develop symptoms of depression.

**Author’s reply:** Thank you for your comments. We have deleted this sentence accordingly.

**Reply to Reviewer #2**

(1) Other confounding factors such as family history, the presence of other comorbid conditions that might predispose to depression and life events need to be considered, especially given the cross-sectional nature of the studies included.

**Author’s reply:** Thank you for your comments. We agreed with the reviewer on that other confounding factors such as family history, the presence of other comorbid conditions that might predispose to depression and life events need to be considered, especially given the cross-sectional nature of the studies included. However, since these factors were generally not reported in the included studies, we could not determine if they may affect the results of the meta-analysis. We have acknowledged this as a limitation of the study in the revised Discussion.

(2) The time used may have been somewhat restrictive.

**Author’s reply:** Thank you for your comments. All studies published from database inception to the last search (December 8, 2023) were screened for possible relevance, which has been clarified in the methods part. With all respect, we believe the time used was not restrictive.

(2) In the introduction, the need to appreciate the bidirectional relationship of diabetes and depression and how this may have influenced the findings reported in the meta-analysis should also have been included.

**Author’s reply:** Thank you for your comments. We have mentioned in the introduction that “Interestingly, subsequent studies suggest that the association between diabetes and depression seems to be bidirectional [9-11]. Besides a high prevalence of depression in patients with diabetes, it is also shown that various measures of depression could be used to predict the risk of type 2 diabetes, such as depression as evidenced by symptom scales, patient diagnosis, face-to-face interviews, and the use of antidepressants [12]”. In addition, we have added some descriptions for the potential bidirectional relationship between prediabetes and depression in the revised Discussion part and to highlight the importance of this hypothesis on the interpretation of the meta-analysis, as “The association between diabetes and depression is considered to be bidirectional. Similarly, it is important to determine if the association between prediabetes and depression is also bidirectional. If the hypothesis is confirmed, this bidirectional relationship could suggest that there may be shared underlying mechanisms linking these two conditions, such as inflammation, hypothalamic-pituitary-adrenal axis dysregulation, and lifestyle factors. Furthermore, understanding this bidirectional relationship can inform future research directions and interventions aimed at preventing and managing both prediabetes and depression”.
