# Supplementary material for: Association between prediabetes and depression: A meta-analysis
Source: PLoS One. 2024 Aug 22;19(8):e0307428. doi: 10.1371/journal.pone.0307428 (PMC11340969; doi:10.1371/journal.pone.0307428)
Supplement: Attachment — Submitted filename: Response letter.docx [file pone.0307428.s002.docx]

Dear Dr. Hennis and the reviewers of Plos One,

Thank you very much for your comments on our manuscript entitled “Association between prediabetes and depression: A meta-analysis” (PONE-D-24-07500R1). These comments are valuable for improving the quality of our work. We have revised the manuscript accordingly, with changes highlighted in red font. A detailed response letter has also been attached for your reference. Your further consideration is highly appreciated.

Look forward to hearing from you at your earliest convenience.

Best regards,

Corresponding author:

Weitao Wan

Department of Psychiatry, Tianyou Hospital Affiliated to Wuhan University of Science and Technology, No. 9, Tujialing, Dingziqiao Road, Wuchang District, Wuhan, Hubei, China. E-mail: wwt686@sohu.com

line 93: ... search terms were based on key words [of - Delete please] rather than MESH terms

**Author’s reply:** Thank you for your comments. This sentence has been revised accordingly.

lines 100-101: Please amend text: studies conducted in adults (18 years and older)....

**Author’s reply:** Thank you for your comments. This sentence has been revised accordingly.

lines 114-115: As a supplementation - this is not grammatical - please amend..

**Author’s reply:** Thank you for your comments. This sentence has been revised as “In addition, the references of related original and review articles were also manually screened for identifying potentially related studies”.

line 121-123: and data collection were independently conducted by two authors. If disagreement occurred, a consultation with the corresponding author was carried out to resolve the disagreement. - There are in fact only two authors, and so following the decision of one author could potentially introduce biases - please clarify.

**Author’s reply:** Thank you for your comments. We apologize for the inaccurate expression in this sentence. In case of disagreement, the two authors discussed it to reach a consensus. This has been revised in the manuscript.

lines 277-278: Please correct 'second' to secondly;

**Author’s reply:** Thank you for your comments. This sentence has been revised accordingly as “Secondly, the meta-analysis protocol was not registered in advance, which could affect the transparency of the methods”

The text 'Not registered prospectively which may influence the transparency of the Methods'.... is not grammatical and needs to be amended;

**Author’s reply:** Thank you for your comments. This sentence has been revised accordingly as “Secondly, the meta-analysis protocol was not registered in advance, which could affect the transparency of the methods”

Please correct 'third' to thirdly.

**Author’s reply:** Thank you for your comments. This sentence has been revised accordingly.

line 292: please correct text to.... 'if they might have affected'

**Author’s reply:** Thank you for your comments. This sentence has been revised accordingly.

line 303: please correct to 'risk factor for depression'...

**Author’s reply:** Thank you for your comments. This sentence has been revised accordingly.
